# Genetic analysis of cryptochrome in insect magnetosensitivity

**DOI:** 10.3389/fphys.2022.928416

**Published:** 2022-08-10

**Authors:** Charalambos P. Kyriacou, Ezio Rosato

**Affiliations:** Department of Genetics and Genome Biology, University of Leicester, Leicester, United Kingdom

**Keywords:** cryptochrome, *Drosophila*, radical pairs, electron spin, quantum, migration, circadian

## Abstract

The earth’s magnetic field plays an important role in the spectacular migrations and navigational abilities of many higher animals, particularly birds. However, these organisms are not amenable to genetic analysis, unlike the model fruitfly, *Drosophila melanogaster,* which can respond to magnetic fields under laboratory conditions. We therefore review the field of insect magnetosensitivity focusing on the role of the Cryptochromes (CRYs) that were first identified in *Arabidopsis* and *Drosophila* as key molecular components of circadian photo-entrainment pathways. Physico-chemical studies suggest that photo-activation of flavin adenine dinucleotide (FAD) bound to CRY generates a FAD^o−^ Trp^o+^ radical pair as electrons skip along a chain of specific Trp residues and that the quantum spin chemistry of these radicals is sensitive to magnetic fields. The manipulation of CRY in several insect species has been performed using gene editing, replacement/rescue and knockdown methods. The effects of these various mutations on magnetosensitivity have revealed a number of surprises that are discussed in the light of recent developments from both *in vivo* and *in vitro* studies.

## Introduction

The magnetosensitivity of migratory birds has long fascinated the biological community with the first demonstration of this phenomenon revealed in the European Robin more than 50 years ago (Wiltschko and Merkel, 1965). It was subsequently discovered that bird magnetic compass navigation was light-dependent and functioned in the blue and green but not red wavelengths (Wiltschko et al., 1993a; Wiltschko and Wiltschko, 1995). However, although the underlying molecular mechanism has proved to be stubbornly elusive, there has been significant progress that can be traced back to the proposal that a class of proteins that were originally identified in plants and animals, the Cryptochromes (CRYs), had the potential to mediate magnetic responses via a radical pair mechanism (RPM) (Ritz et al., 2000). The first CRY-encoding genes to be isolated were the blue light photoreceptors in *Arabidopsis thaliana, AtCry1* (Ahmad and Cashmore, 1993) and *Drosophila melanogaster, dcry* (Emery et al., 1998; Stanewsky et al., 1998). Both molecules were shown to be intimately involved in circadian light responses, in plants and flies respectively (Cashmore et al., 1999). Putative CRYs were also identified in humans and initially considered blue light responsive (Hsu et al., 1996).

Sequence comparisons revealed that the CRYs are evolutionary related to the photolyases, which represent an ancient class of enzymes that repair UV-induced DNA damage. The major difference between these two protein classes is in the extended C-terminal that is characteristic of the CRYs and plays a prominent role in both light and magnetic responses (Cashmore et al., 1999; Rosato et al., 2001; Fedele et al., 2014a; Fedele et al., 2014b). CRYs are ubiquitous and found in all branches of life and are part of a large gene family with at least six subgroups in metazoans (Haug et al., 2015). Animal CRYs can be divided functionally into three main classes. The first two encompass the *Drosophila*-like type 1 and the bird specific type 4 CRYs that are photosensitive. The third corresponds to the vertebrate-like type 2 CRYs that in many animals (including birds and insects) act as repressors that negatively regulate the circadian clock and may not be directly responsive to light. The circadian mechanism is an autoregulatory transcription/translation feedback loop that revolves around the rhythmic expression of its positive and negative regulators (reviewed in (Ozkaya and Rosato, 2012). The type 2 CRYs function as negative regulators together with Period (PER) proteins. However, some animals, such as *Drosophila*, have lost type 2 CRYs so instead they use Timeless (TIM) in partnership with PER as circadian repressors (Yuan et al., 2007; Kotwica-Rolinska et al., 2022).

### The radical pair model/mechanism

**FIGURE 1:**
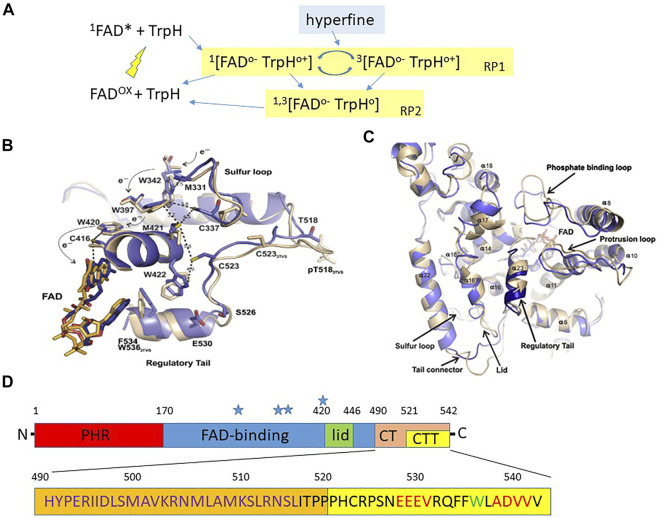
Structure of *Drosophila* cryptochrome.**(A)** The framework for a CRY photocycle using *Escherischia coli* photolyase (*Ec*PL) and *Arabidopsis thaliana* CRY1 (*At*CRY1). Photoexcitation excites the oxidised FAD (FAD^ox^) to the singlet state (^1^FAD*) which then receives an electron via a chain of three Trp residues (see Figure 1B) within CRY/photolyase generating a radical pair leaving the terminal Trp minus an electron ^1^ [FAD^o−^ TrpH^o+^] in the singlet state with antiparallel electron spins which can either reverse to the ground state (FAD^ox^ + TrpH) or interconvert to the triplet state ^3^ [FAD^o−^ TrpH^o+^] *via* hyperfine interactions in which the electron spins of the unpaired electrons are in parallel. In *Ec*PL the S and T forms of this RP (RP1) can also convert to a second RP2 by deprotonation (removing H ion) of TrpH^o+^ to the neutral radical, Trp^o^ which can return to the dark resting state (FAD^ox^ + TrpH) by further redox reactions (redrawn with amendments from Sheppard et al., 2017). **(B)** Crystal structure of *d*CRY regions lying close to the FAD (blue from Czarna et al., 2013, beige Zoltowski et al., 2011). The Trp triad W420, 397 and 342 are shown together with the proposed electron skipping that generates the photoreduced FAD-Trp radical pair. **(C)** Crystal structure of *d*CRY from Czarna et al (2013). The blue represent the structure from Czarna et al. compared to a previous structure in beige from Zoltowski et al (2011). There are some significant differences in the tail structure, later corrected by Zoltowski et al (2011). **(B,C)** reproduced with permission from Czarna et al. (2013). **(D)** Overview of *d*CRY landmarks (not to scale). PHR, Photolyase homology domain; CT, C terminal residues 490–542; CTT C terminal tail, residues 520–542; Blue stars indicate positions of Trp tetrad, Trp 342, 394, 397 and 420. The amino acid sequence of the CT is illustrated below. The calmodulin binding domain is shown in orange, residues ∼490–516 in violet. PDZ domain binding motifs in the CTT are in red, and the sole Trp in the CTT is green. The *dCRYΔ* transgene is missing the CRY CTT and the *GFP-CRY CT* transgene encodes GFP fused to residues 490–542 (see text).

That radical pairs (RPs) could be sensitive to magnetic fields was proposed by Schulten et al. (1978). The formation of radicals requires energy provided by light that puts into motion an electron transfer from a donor (which, may be organised into a chain) to the excited acceptor molecule. Thus, the donor is left with one less electron and a positive charge, whereas the acceptor has one extra electron and a negative charge, resulting in two paired radicals. Paired electrons can spin in opposite antiparallel (singlet state, S) or in parallel directions (triplet states, T). The interconversion between S and T states is facilitated by the magnetic interaction with nearby atomic nuclei, called the hyperfine coupling, which produces a rapid S to T interconversion (Figure 1A) (Ritz et al., 2009). We can fancifully imagine that the oscillation between S and T states is similar to an elephant precariously and finely balanced on a ball, which can easily flip to the left or to the right. While an elephant standing on the ground cannot be moved easily, the one on the ball only needs a tiny push for it to fall. Using this (ludicrous) analogy, a tiny force (push) such as the earth’s ∼50 μT magnetic field (MF) can alter the dynamics of the unstable S to T interconversion (the elephant falling left or right) favouring one or the other spin state. This affects the half-life of the RP and of the associated conformational changes of the molecule, with corresponding downstream effects (Hore and Mouritsen, 2016).

*D. melanogaster* and *A. thaliana* CRYs are flavoproteins and have a FAD (flavin-adenine dinucleotide) binding site to which this catalytic chromophore attaches itself (Figure 1B). A second light-harvesting bound chromophore, pterin, that transfers energy on light activation to FAD is found in *Arabidopsis At*CRY1 (Hoang et al., 2008), but not apparently in Drosophila *d*CRY (Selby and Sancar, 2012). In *d*CRY, photoexcitation of FAD in its resting oxidised state by blue light (Figures 1A,B), generates electron transfer across a triad or tetrad (see below) of Tryptophan (Trp) residues (Sheppard et al., 2017). The FAD acceptor ends up with one extra electron (FAD^o−^ the semi-reduced flavosemiquinone radical) whereas the terminal Trp donor is left minus one electron Trp^o+^, thereby generating the magnetically sensitive RP. From the structure of CRYs the three or four relevant Trps in dCRY are Trp342, 394, 397 and 420, with Trp342 or Trp394 representing the critical terminal residue that generates the radical pair with FAD (Figures 1A,B). The transfer of the electron from FAD^o−^ back to the terminal Trp^o+^ to return to oxidised FAD can only occur from the S state (Hore and Mouritsen, 2016). In CRYs, the earth’s MF pushes the S-T interconversion towards the T state, causing the RP to persist for longer. Further light absorption fully reduces FAD^o−^ to FADH^−^ that can be converted back to the oxidised form in darkness by O_2_ (Hore and Mouritsen, 2016). This mechanism of reoxidation can generate other radical pairs, which in principle, may be sensitive to a MF even in darkness (Müller and Ahmad, 2011).

Much of the evidence for the existence of RPs within CRYs is based on *in vitro* spectroscopic analyses so it is important to consider whether these physico-chemical and often theoretical approaches can be supported *in vivo.* Assuming the RPM provides the fundamental quantum basis for magnetosensitivity what else would be required to make CRY the cognate receptor for avian navigation? Given the necessary photobiology, CRY should be associated with an organ that is exposed to light and the eye is the obvious candidate. Indeed, the first suggestions of radical pairs in magnetosensitivity were focused on rhodopsins (Hong, 1977; Leask, 1977). A second requirement would be that the RP of CRY should be sensitive to the inclination of the earth’s magnetic field as that is the geophysical feature that provides navigational cues (Wiltschko et al., 1993b). One way that might occur is by CRY having a fixed orientation within the containing cells (presumably neurons so that they can generate a behavioural output) and these being spatially organised in a certain fixed orientation within the eye. As the bird turns its head (and its eyes), changes in the orientation of the MF with respect to the bird’s body axis alter the spin dynamics of the RP that allows the bird to compensate its direction of flight. Ritz et al. (2000) provide an interesting early theoretical model and simulation of how this might work. How birds might ‘see’ the magnetic compass lines by dipole interactions within the retina between the RP generating molecule and the opsins has also been discussed (Stoneham et al., 2012).

A RPM underlying navigation is difficult to study in birds because, unlike model organisms such as *Drosophila* they are not easily amenable to molecular and genetic analyses. However, flies are not known for their prowess in navigation and migration so could they provide a model system that is useful to study the RPM and be acceptable to ornithologists and physical-chemists? First, consider that the putatively ‘relevant’ CRY of the night-migratory European Robin, *Erithacus rubecula* (ErCRY4), shares the relative positions of the four Trps that theoretically mediate the formation of the RP, with both *D. melanogaster* CRY (*d*CRY), and that of the long-distance migrant the Monarch butterfly *Danaus plexippus* (*Dp*CRY1)(Xu et al., 2021). Second, it has been demonstrated repeatedly, by several different laboratories using various paradigms that *Drosophila* and Monarch respond behaviourally to MFs (see later section). Third, while the behavioural output of MF experiments is very different in birds, Monarch and *Drosophila*, given the conservation of the four RP-related Trps in CRYs, the fundamental quantum spin chemistry underlying magnetosensitivity is likely to be the same in these species whereas the downstream effectors of the CRYs may be different according to species and even cell type. If we accept these premises, then insects such as *Drosophila*, which have the most sophisticated molecular genetic toolkit available, can provide a rigorous and critical experimental analysis of the RP model.

### Molecular and structural basis for CRY photoactivation and magnetoreception

While we have discussed the quantum biology and spin dynamics underlying the RPM we have not introduced the molecular/biochemical/structural basis for CRY-mediated magnetosensitivity. Figures 1B,C illustrate the important structural features of CRY (Czarna et al., 2013) relevant to photo and magnetoreception and Figure 1D shows the overall architecture of *d*CRY based on structural, functional and bioinformatics analyses (Rosato et al., 2001; Hemsley et al., 2007). The N-terminal corresponds to the photolyase homology region (PHR) followed by the more central FAD binding region (Figure 1D). Towards the C-terminal (CT, residues 490–542) there is a lid (residues ∼420–446) and the tail (CTT, residues 521–542). The C-terminal tail (CTT) that is absent in the *d*CRYΔ mutant (discussed below) distinguishes *d*CRY from the photolyases in that it largely replaces the latter’s DNA substrate bound to the FAD pocket (Zoltowski et al., 2011; Czarna et al., 2013; Levy et al., 2013). Without the CTT, dCRYΔ is unstable because it adopts the active (“open”) conformation in both light and dark, which makes it constitutively prone to signalling and to degradation (Rosato et al., 2001). Possibly, such instability is due to the unrestricted access by BRWD3 (part of the CRL4 E3 ubiquitin ligase complex) to the FAD pocket, resulting in continuous ubiquitination and proteasomal degradation (Ozturk et al., 2013; Kutta et al., 2018). Within the CTT there are two putative PDZ domain binding motifs and a Trp536 residue. A calmodulin (CaM) binding site is also predicted within a region (residues 490–516) that is a hotspot for protein-protein interactions (Rosato et al., 2001; Hemsley et al., 2007; Mazzotta et al., 2013).

This linear representation of *d*CRY in Figure 1D conceals the folded nature of the protein which is revealed in structural studies showing that the PHR FAD, lid, CT and CTT all lie in close proximity (Figures 1B,C) (Zoltowski et al., 2011; Czarna et al., 2013; Levy et al., 2013). Photoinduction of FAD generates the RP through electron-skipping and mutations Trp397Phe and Trp420Phe obliterate FAD^o−^ formation (Czarna et al., 2013). Mutational analysis of other residues close to each of Trps 420, 397, 342, namely Cys416, Cys337 and Met331 reveals that they are involved in the gating of the electron transfer. In the CTT, Cys523 is important for FAD photoreduction and Phe534, Glu530 and Ser526, anchor the tail to the PHR region in the dark state whereas Ser526 may act as a hinge for the CTT. Consistent with this is the effects of a Ser526Ala mutation that prevents the light degradation of dCRY (Czarna et al., 2013) and the physical interaction of *d*CRY with TIM and PER (Hemsley et al., 2007). Light induced FAD photoreduction displaces the CTT allowing TIM (acidic) and Jetlag (JET, basic, involved in CRY-dependent, light-mediated degradation of TIM; Peschel et al., 2009) to associate with acidic and basic regions adjacent to the CTT. This explains mechanistically why *d*CRYΔ and an almost identical *d*CRY^M^ mutant that are both missing the CTT are constitutively active in both light and dark (Rosato et al., 2001; Busza et al., 2004; Dissel et al., 2004). The CTT therefore prevents the association in the dark of *d*CRY with the key factors TIM and JET, which is necessary for circadian light responses. Figure 1B shows the location of these functionally important *d*CRY residues in relation to the FAD.

Photoactivation of CRY is a critical step for both circadian entrainment in insects and in RP biology and this relies on bound FAD. Given that Type 1 CRYs are photosensitive and Type 2 apparently not, what is the evidence that FAD binds each CRY type? This is particularly relevant in the debate about whether type 2 circadian repressor CRYs are magnetosensitive, with some studies showing that they are while others that they are not. When purified, dCRY binds to FAD in its oxidised form and on blue light exposure converts to the FAD^o−^ radical (Berndt et al., 2007). The crystal structure of *d*CRY reveals the absence of the characteristic residues to bind pterin (an additional cofactor bound to 6-4 photolyases) in contrast to the well-conserved FAD binding centre (Czarna et al., 2013). The corresponding FAD domain in mouse CRY1 (there are two type 2 CRYs, confusingly, mCRY1 and mCRY2) is also present, so theoretically it can bind FAD. This is in contrast to a study in which mammalian CRYs appeared unlikely to bind physiologically relevant quantities of FAD, as the binding pocket would be too open to ‘hold’ the cofactor for long enough and this would result in constitutive activation of the protein (Kutta et al., 2017). A further study has introduced another twist, revealing that FAD stabilises mammalian CRYs (Hirano et al., 2017). Ubiquitin ligase FBXL3 binds to the FAD pocket, so competition between cofactor and FBXL3 binding modulates CRY stability and alters the kinetics of CRY ubiquitination and subsequent degradation (Xing et al., 2013). A similar mechanism has been suggested for *d*CRY, with BRWD3 having a similar function to mammalian FBXL3 (Ozturk et al., 2013; Kutta et al., 2018). Interestingly, the first mutant identified in *Drosophila*, *cry* ^*b*^ *,* revealed a missense mutation in the FAD binding domain resulting in a highly unstable protein (Stanewsky et al., 1998). Additionally, the protracted light activation of *d*CRY seemingly results in the irreversible opening of the FAD pocket and the expulsion of the cofactor, making activated *d*CRY quite similar in structure to mammalian type 2 CRYs (Kutta et al., 2018). Thus, for both type 1 and 2 CRYs, FAD binding is associated with stability rendering the two types more similar than previously believed. Perhaps then, their photo- and magnetosensitivity differences are more quantitative than qualitative so that biologically relevant quantities of FAD may bind in an appropriate cellular environment such as an insect cell.

## Genetic analysis of magnetic phenotypes in *Drosophila*

### Training and testing phenotypes

A demonstration of a magnetic sense in *Drosophila* had flies trained under a wavelength of light of 365 nm in an ambient field facing north and tested in an 8 arm radial maze spanning each point in the compass (Phillips and Sayeed, 1993). Male (but not female) flies showed a strong tendency to choose the northern pointing arm of the maze. Under a green 500 nm light source, this preference was shifted by 90^o^ to the east. These experiments revealed that the magnetic sense can be stimulated by light in the UV/blue to the green wavelengths which fits with the spectral characteristics of *Drosophila* opsins, particularly Rh5 and Rh6 as well as dCRY (VanVickle-Chavez and Van Gelder, 2007; Sharkey et al., 2020).

Subsequent genetic analyses employed flies that were trained in a T-maze by associating a sucrose reward with exposure to a magnetic field (Gegear et al., 2008; Gegear et al., 2010). Under full spectrum light, naïve flies tend to avoid a MF, but after training, they modestly (but significantly) preferred to enter the arm that was exposed to the MF. This behavioural effect could be demonstrated up to a wavelength of 420 nm, which matches the action spectrum of dCRY, and *cry* mutants were defective in this trained magneto-response. A follow-up study revealed that *cry-null* mutant transgenic flies carrying the *D. plexippus* Monarch *Dpcry1* and *Dpcry2* genes could also be trained to move towards a MF up to a wavelength of 420 nm. Furthermore, mutating (what was considered at the time) the terminal Trp to phenylalanine (Phe) that cannot generate a RP in both *Drosophila* dCRY (Trp342Phe) and Monarch *Dp*CRY1 (Trp328Phe) did not disrupt the normal magnetoresponse but did so for the terminal Trp in Monarch *Dp*CRY2. As these mutations should prevent the electron skipping that generate the RP, the results cast some initial doubt on the canonical RP model. However, since these studies, the possibility has been raised that there is a fourth terminal Trp (which would be Trp394 in *d*CRY) that would remain intact in these *d*CRY mutants (Muller et al., 2015; Nohr et al., 2016). However, its role in magnetosensitivity, either as the terminal Trp or as a functional partner to Trp342 remains to be determined (Muller et al., 2015; Nohr et al., 2016; Wong et al., 2021).

One of the surprising results from these experiments is that *D. plexippus Dp*CRY2 also gave a magnetic response in transgenic flies. Using the same assay, human *h*CRY2, which is normally highly expressed in the human retina (Thompson et al., 2003), was also shown to be magnetosensitive in transgenic *Drosophila* (Foley et al., 2011). As CRY2s are not generally believed to be light-sensitive (despite Hsu et al., 1996) it might suggest the possibility that other light-sensitive molecules in the transgenic fly, such as the fly opsins, might be priming CRY2s to mediate the magneto-response, in effect transferring the light signal to CRY2s.

### Circadian-based phenotypes

**FIGURE 2:**
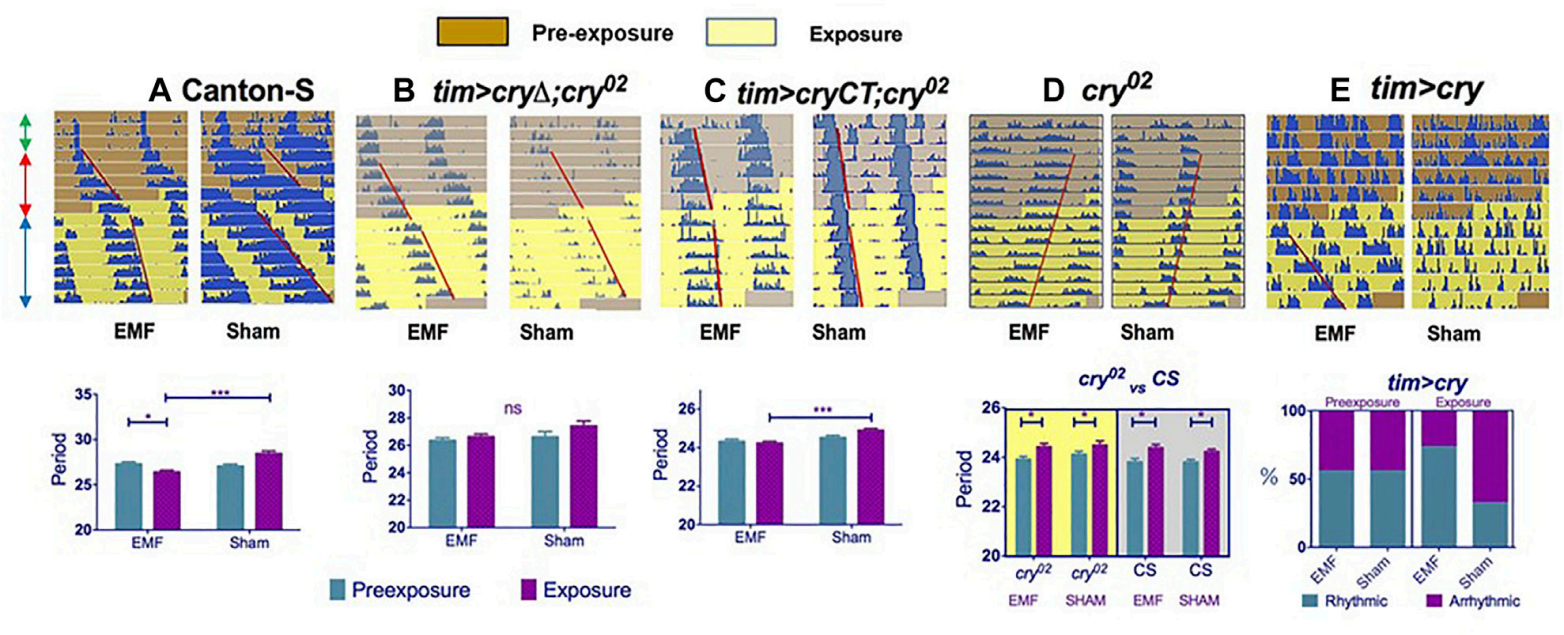
Exposure to a low frequency magnetic field has a circadian phenotype in *Drosophila*. Top row **(A**–**E)**: double plotted representative actograms from a male fly from each genotype. The left hand actogram for each genotype shows the activity of a fly exposed to a MF, whereas the right hand panel shows the activity under sham exposure. Each row of the actogram shows 2 days of activity day1 and day2, below which is represented day2 and day3, then day 3 days 4 and so on. The double headed green arrows on the left of panel A show the first 3 days in a dim blue light-dark BLD12:12 cycle, followed by 5 days in constant dim blue light, BLL (prexposure, red arrows, brown background on actogram), followed by 8 days in the same BLL conditions but with exposure (or sham) to a 3 Hz, 300 μT MF (blue arrows, yellow background). Red lines show offsets of free-running locomotor activity reflecting any change in period after exposure. **(A**–**C)** The graphs below show the corresponding mean free-running period and sems for each genotype calculated from the pre-exposure and exposure conditions in the sham and experimental groups. **(A)**: Canton-S wild-type **(B)**: *tim > cryΔ;cry* ^*02*^ (*timGAL4; UAScryΔ;cry* ^*02*^) which means CRY that is missing the CTT (last 20 amino acids) is expressed only in clock cells in a *cry-null* background. **(C)**: *tim > CRYCT;cry* ^*02*^ (*timGAL4; UAS-GFP-CRYCT;cry* ^*02*^) which expresses only the 52 amino acid CRYCT (fused to GFP for stability) in clock neurons (*tim > CRYCT;cry* ^*02*^) in a *cry-null* background. **(A,C)** show significant period shortening under exposure (but not sham), whereas the MF has no significant effect on period for the *cryΔ* transgene (see Figure 1). **(D)** The *cry* ^*02*^ null mutant shows no changes in period on exposure to a MF compare to sham (upper panel). The lower panel reveals that the response of *cry* ^*02*^ flies in constant dim BL (shown as actograms in the upper panel and on a yellow background in the lower panel) is no different from the response of *cry* ^*02*^ mutants in constant darkness (gray background in lower panel). Consequently by not showing any period lengthening under BL, *cry* ^*02*^ mutants are not informative in this assay. **(E)** In contrast to *cry* ^*02*^ *,* overexpression of CRY in clock neurons (*tim > cry*) in a wild-type background leads to high levels of arrhythmicity in the prexposure conditions, but application of a MF significantly rescues this arrhythmicity (upper and lower panels). Furthermore, rhythmic flies on exposure to a MF have shorter periods than those rhythmic flies that are exposed to sham (not shown, see Fedele et al., 2014a). Figure 2 redrawn from Fedele et al. (2014a).

*D*CRY is considered a dedicated circadian photoreceptor in *Drosophila*, so an attempt was made to observe MF effects on free-running circadian locomotor activity cycles (Yoshii et al., 2009). Constant dim blue light (470 nm) that was not intense enough to cause arrhythmicity was used to lengthen free-running circadian period providing a ‘sensitised’ background on which a static MFs could be applied. The results were quite variable with 50% of the flies showing no effect but of those that did respond to the MF (300 μT), the majority lengthened their free-running period. The net change in period irrespective of direction (longer or shorter) was greater for the exposed flies compared to controls (0 μT). When repeated in red light there was no difference in net period change between experimental and control groups. The use of the nearly null *cry* ^*b*^ mutant was not informative as these mutants do not ‘see’ the light so their period does not lengthen under constant dim blue light - they simply free-run as if in constant darkness (DD) with no period changes on exposure to the MF (see also Figure 2D). A more relevant result was obtained when dCRY was overexpressed in clock neurons. Most flies were arrhythmic in dim blue light, but of those few that were rhythmic, the net change in period was greater than that of wild-type. These results suggested that dCRY might be involved in the fly’s magnetic response.

Given that the effects of Yoshii et al. (2009) were quite modest, an attempt to replicate these findings was made using state-of-the-art exposure chambers, a constant dim blue light at 450 nm that lengthened free-running period and exposure to static/low-frequency fields (0, 3 and 50 Hz) under different field intensities (90, 300, 1000 μT) (Fedele et al., 2014a). In all these experiments, although the free-running periods were much longer than 24 h, flies exposed to a MF nevertheless showed a consistent reduction in period length compared to sham controls (Figure 2A). These results were in contrast to those of Yoshii et al. (2009) who generally observed that most flies exposed to a MF tended to have longer periods than sham controls. To examine further this apparent discrepancy, the experiments were repeated under green light at 500 nm, which revealed the opposite phenotype to blue light, namely a further significant period lengthening under MF exposure when compared to sham. This blue-green difference in magnetic responses has been noted several times by Phillips and collaborators in different species and is interpreted as reflecting an antagonistic effect at the two wavelengths (Phillips et al., 2010). Thus, the 470 nm wavelength used by Yoshii et al (2009) would mix both the blue (reduced period length compared to sham) and the green (increased period length) circadian MF responses, almost cancelling out any period phenotype.

Fedele et al (2014a), in agreement with Yoshii et al. (2009), also observed that overexpression of dCRY in clock neurons gave high levels of arrhythmicity under blue LL, consistent with the known role of CRY. However, on exposure to the MF, many of the same flies that were previously arrhythmic in LL now became rhythmic (Figure 2E), as if the MF exposure had suppressed the normal response of CRY to light (Fedele et al., 2014a). This same interpretation, can explain the shorter periods under the MF in blue light (Figure 2). When dCRY is activated by light it drives a longer circadian period and arrhythmicity (depending on light intensity and duration) as well as its own degradation. Perhaps the MF reduces the normal light response, which in dim constant LL, should give a longer and longer period that eventually merges to arrhythmicity? Indeed, under a MF in constant blue LL, dCRY extracted from fly heads appears to be more stable than under sham MF, supporting the view that the MF is somehow curbing the normal CRY-mediated light degradation response (Fedele et al., 2014a). Further experiments revealed that the Trp342Phe mutation of the putative ‘terminal’ Trp did not prevent the circadian MF response, mirroring the results of the T-maze conditioning assay described above. However, the most unexpected result was observed when a ∼50 residue fragment of the CRY C-terminal (CT) was expressed in a *cry-null* background. This transgene has the sequences encoding the CRYCT fused to those for GFP for stability (*GFP-CT*) and does not carry the four canonical Trps of the electron chain nor the FAD binding domain implicated in the RP mechanism. Nevertheless, and quite remarkably, this GFP-CT fragment was sufficient to mediate the circadian MF response when expressed in clock neurons (Figure 2C)(Fedele, et al., 2014a)

A second phenotype also emerged from the locomotor behaviour analysis. The levels of activity were altered under MF exposure so that wild-type flies became hyperactive. Intriguingly, flies carrying a *dcry* transgene missing the C-terminal tail (CTT) of ∼20 residues (*dCRYΔ*)*,* did not show circadian-period shortening under MF exposure, but were nevertheless hyperactive, whereas the GFP-CT encoding transgene that produced period-shortening, did not generate hyperactivity. A similar hyperactivity was found for flies carrying the *hCry2* (but not the *hCry1*) transgene. Consequently, the N-terminal region of *d*CRY that contains the four Trps and the FAD binding domain can mediate MF induced hyperactivity but not the period shortening, with the opposite phenotypes generated by the CRY C terminal (GFP-CT) (Fedele, et al., 2014a). *h*CRY2 has a similar N-terminal to *d*CRY but a very different C-terminal, so there exists a correlation between the sequence composition of the CRYs N- and C-terminals and the MF phenotypes observed.

### Geotaxis phenotypes

**FIGURE 3:**
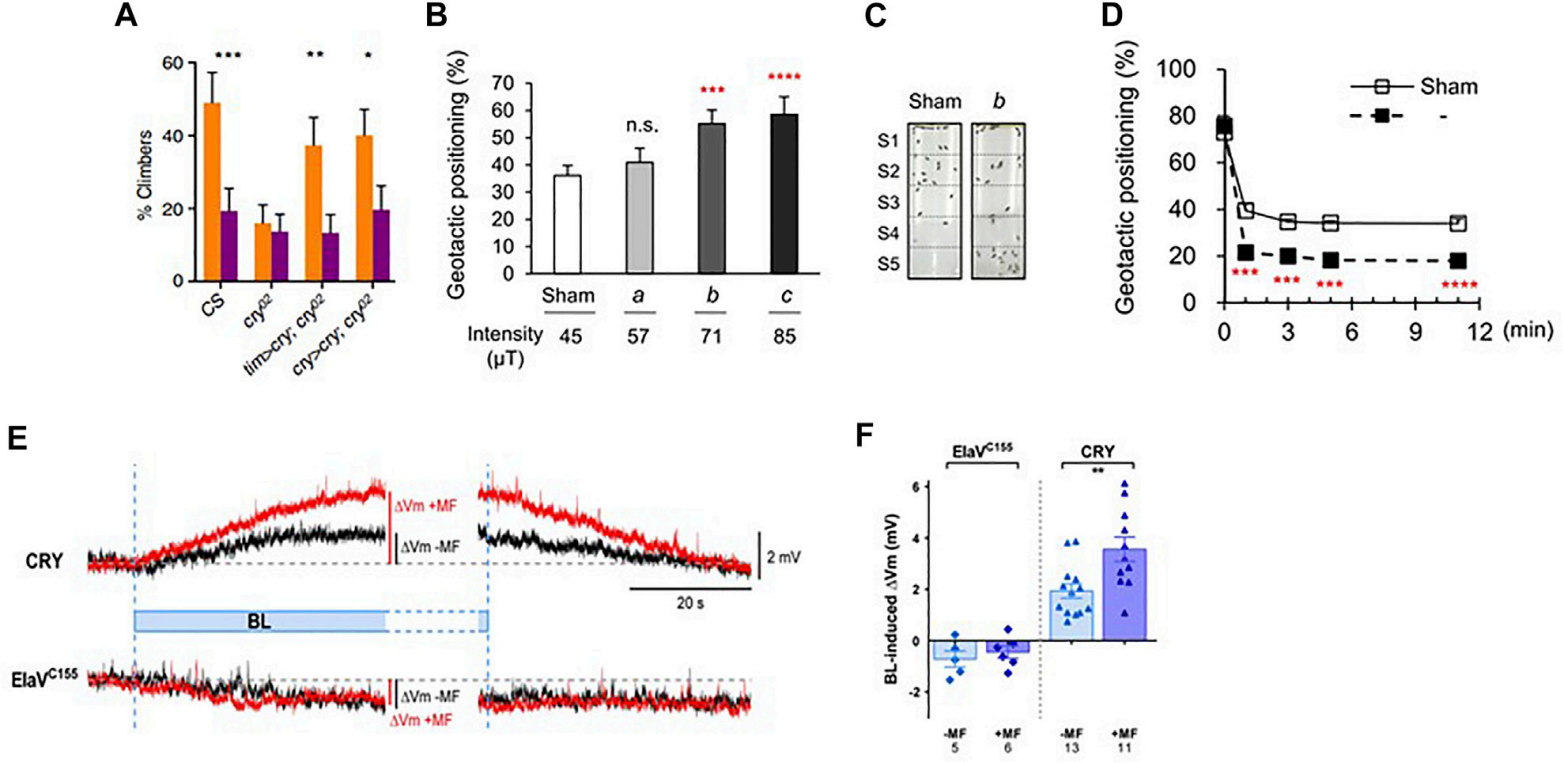
MF exposure effects on geotaxis and neuronal firing in *Drosophila*. **(A)** Results of geotaxis assay from Fedele et al. (2014b). Under 450 nm blue light wild-type Canton S (CS) flies walk upwards (negative geotaxis), but application of a MF makes them more positively geotactic generating a significantly lower climbing score as they tend to move downwards. *cry* ^*02*^ mutants show reduced negative geotaxis, so application of a MF does not change their behaviour, therefore as in circadian behaviour (Figure 2D) this result is not informative with respect to the MF. However, expressing CRY in a mutant *cry* ^*02*^ background either in all clock neurons (*tim > cry*) or just those that normally express CRY (*cry > cry*)*,* rescues the normal negative geotactic response in sham exposed flies which is significantly reduced in MF exposed flies. Means and sems shown. Flies were exposed to a static 500 μT MF in dim blue light.*, *p* < 0.05, **p < 0.01, ***p < 0.001. **(B,C)**. Results of similar experiment as in A by Bae et al (2016). Geotactic positioning (positive geotaxis) in a tube, very similar to that used by Fedele et al., 2014b (see above, A) and shown in C, at different MF intensities. The graph shows means and sems for flies exposed to the earth’s ambient field (sham, 45 μT in Korea) with increasing intensity of exposure. In this case, the results are represented as the flies’ positive geotaxis score (% flies in lower S2-S5 sections of tube shown in C, so higher scores means the flies are moving further downwards). As field intensity increases, flies move further downwards generating a higher geotactic score, as observed in Fedele et al, 2014a)*.* ***p < 0.0005, ****p < 0.0001 compared to sham. **(D)** Geotaxis in a near zero field (Bae et al., 2016). Geotaxis was compared in a zero (labelled ‘b’ see C) versus ambient 45 μT (sham) field. The ambient (sham, 45 μT) field significantly enhanced positive geotaxis (open squares) compared to zero field (filled squares), so flies moved higher in zero field, in contrast to the downward (positive geotactic) effect of the ambient MF on geotaxis. ***p < 0.0005, ****p < 0.0001. Figures **(B–D)** reused under open access creative commons license (http://creativecommons.org/publicdomain/zero/1.0/). **(E,F)** Magnetic field effects on neuronal firing in *Drosophila* larval aCC neuron (reproduced from Figure 1 in Giachello et al., 2016 under creative common license). **(E)** Representative electrophysiological recordings from a larval aCC neuron expressing CRY ectopically by using the pan-neuronal *elavgal4* driver driving *UAS-dcry* (labelled ‘CRY’) compared to control (*elavGAL4* driver only). Blue light produces significant reversible depolarization in membrane potential (ΔV_m_, Y-axis, black trace) that is potentiated by a 100 mT MF (red trace). The *elavgal4* driver control generates an increased depolarisation to BL (black) but no enhancement by the MF (red). **(F)** The results from a group of larvae are shown. Means and sems **p < 0.01.

*D. melanogaster* are negatively geotactic and, when disturbed, walk upwards against gravity rather than walking downwards (positive geotaxis). Negative geotaxis, like most behaviour, is influenced by many genes, and an early microarray analysis revealed that genetic variation in two circadian-related loci, *cry* and *Pdf* [*Pigment dispersing factor,* encoding a neuropeptide that allows clock neurons to communicate with each other (Renn et al., 1999)] could contribute to the geotactic response (Toma et al., 2002). Thus, it was of obvious interest to examine whether this phenotype was sensitive to MFs. Flies showed reduced negative geotaxis under a MF in blue, but not red light (Fedele et al., 2014b) (Figure 3A). Under a sham MF *cry* ^*02*^ null mutants did not climb upwards, so not surprisingly (and uninformatively), exposure to a MF had little effect. However, when *d*CRY was expressed with either the *timGAL4 or cryGAL* drivers a normal negative geotaxis was generated under sham, which was reduced on exposure to a MF (Figure 3A). It was also observed that expressing *d*CRY in clock neurons, or eyes or antennae could rescue the geotactic MF response. In contrast, using mutations to remove the eyes or the antennae, disrupted the MF response. These results suggest that at least one of these three tissues, eyes, antennae or clock neurons must express *d*CRY in the presence of intact structures of the other two, in order to mediate the geotactic response to MFs. Furthermore, neither *hCry1* nor *hCry2* could rescue the MF phenotype in a *cry-null* mutant background.

A similar independent but far more sophisticated analysis of fly geotaxis from Chae’s group in Korea replicated and significantly extended the work with *d*CRY outlined above, using both a similar tube positioning assay as Fedele et al. (2014b), a vertical choice geotactic Y-maze and free-flight recordings under blue light (Bae et al., 2016) (Figures 3B–D). Under near 0 MF, flies became more negatively geotactic in both maze and flight tests and exposing them to a MF reduced negative geotaxis (i.e., geotaxis became more positive with flies moving downward under a MF). A task was also employed in which food was associated with positive geotaxis in the training condition under a 0 MF. Testing with no food in 0 MF, flies subsequently showed a positive geotactic rather than the normal negative geotactic score. Using various mutants it was also revealed that dCRY, PDF and PYREXIA (PYX) were involved in the geotactic MF response and rescue experiments using *Gal4/UAS* in mutant backgrounds further revealed that *dcry* and *pyx* were required in the antennae to mediate the MF responses, confirming the earlier antennal results of Fedele et al (2014b). The results from both groups suggested that the Johnston’s Organ (JO) was the important antennal mechanoreceptor for generating the MF-modulated geotactic responses. Bae et al (2016) also showed indirectly, using a *cryGAL4* driving *UASGFP*, that *d*CRY was expressed in JO, but found no evidence for PDF expression. Yet knocking down PDF in *cry*-expressing cells disrupted the geotactic MF response, suggesting that the geotactic information from JO converges on neurons that express CRY and PDF, namely the (s-LNvs) and large ventral lateral neurons (l-LNvs), two prominent groups of clock cells (Yoshii et al., 2008). This result fits nicely with the work of Fedele et al (2014b) in that the peripheral CRY-expressing MF sensing organs, JO and eyes, require the clock cells (with or without CRY expression) to mediate the MF geotactic phenotype.

A second remarkable study from the Korean group examined ‘geomagnetic imprinting’ in flies in which developing embryos, larvae and pupae were exposed at different points in their life cycle to a gradient MF that was characteristic of their location in Korea (Oh et al., 2020). Emerging adult flies that were starved and had been pre-exposed to the local MF as embryos, 6–9 h after egg laying, showed more positive geotaxis (moving downwards) to the applied MF gradient compared to control conditions (sham or reversed gradient). Replacing the gradient MF exposure to one characteristic of Vancouver or Madrid had the same effect, but mixing and matching different geographical exposures in the pre-exposure and testing regimes had little effect on geotaxis. In other words, the embryos had become ‘imprinted’ on the pre-exposure MF gradient. Even more surprising was that these MF-induced effects proved to be transgenerational. The progeny of flies pre-exposed to MFs showed similar geotactic scores to the parents, even though they had not been further pre-exposed to a MF. This downward phenotype required both the male and female parent to have been previously pre-exposed, implying some form of epigenetic inheritance.

In these studies it was starved (but not satiated) flies that reveal these MF-induced geotactic responses suggesting that the phenotype is foraging-relevant. The positive geotactic response under a MF may provide a way for flies to forage more successfully, given that this species preference is for rotting fruit that is located mainly on the ground. The authors also speculate that imprinting might provide a way by which flies may return to a food/mating site from which they successfully emerged, although the ephemeral nature of food sites makes this a bit of a stretch. Nevertheless, these intriguing studies from the Chae group generate some possible answers to why flies might have a functional magnetic sense.

### Other MF phenotypes in *Drosophila*

*Drosophila* larvae convulse if they have been previously stimulated 11–19 h into embryogenesis with pulsed blue (470 nm) light, but not orange (590 nm) light or darkness (Marley et al., 2014). Recovery time from these seizures is dramatically lengthened by exposure to a 100 mT MF and this phenotype is ameliorated in *cry-null* mutants and by anti-epileptic drugs. *d*CRY is optogenetic, so when activated by blue light it generates neuronal firing via changes in the permeability of potassium channels (Fogle et al., 2011; Fogle et al., 2015). This provides a potential explanation for the larval seizure phenotype through increased blue-light stimulated *d*CRY-mediated synaptic activity, which is further modulated by the effects of the MF. Furthermore, expressing *d*CRY ectopically in the larval aCC motor neuron stimulates action potentials under blue light, which is further potentiated by exposure to a 100 mT MF (Giachello et al., 2016) (Figures 3E,F). Once again, the *Trp342Phe* mutation did not compromise the MF effect, in contrast to the *dcryΔ* C-terminal tail deletion, so in this assay both mutants mirrored their circadian period MF phenotype. Several other *dcry* mutants have now been tested in both circadian behaviour and larval physiology and the results reveal parallel effects in the two paradigms (Bradlaugh et al., 2021). While behaviour provides a whole organism readout of responses to a MF, single cell neuronal physiology is several biological steps closer to the initial quantum changes that initiate the MF molecular cascade. It is therefore comforting that the adult fly’s behaviour and the single larval neuron show such correlated magnetic responses to *d*CRY manipulations.

Larval behaviour has also been investigated in a crawling assay on an agar covered Petrie dish. Larvae will avoid an area that is exposed to a MF under blue but not red light. Mutant *cry* ^*b*^ or *cry* ^*02*^ larvae do not avoid the MF showing this phenotype is CRY-dependent (Sherrard et al., 2018). In this assay *h*CRY1 can rescue the mutant MF avoidance phenotype, further suggesting that in *Drosophila* vertebrate-like CRY2s can be blue-light sensitive, directly or indirectly. However, the heterogeneous response of CRY2s in rescuing a *dcry*-*nul*l background in different behavioural assays discussed so far points to the role played by the cellular context in affording light perception or downstream signalling capabilities to these molecules.

In summary, there is a list of phenotypes in *Drosophila* that respond to MFs that in adults includes, association tasks, circadian cycles, geotaxis, male courtship (Wu et al., 2016), and in larvae, seizures, MF avoidance and synaptic responses, all of which are blue light and CRY-dependent.

### MF effects and genetics in other insects

**FIGURE 4:**
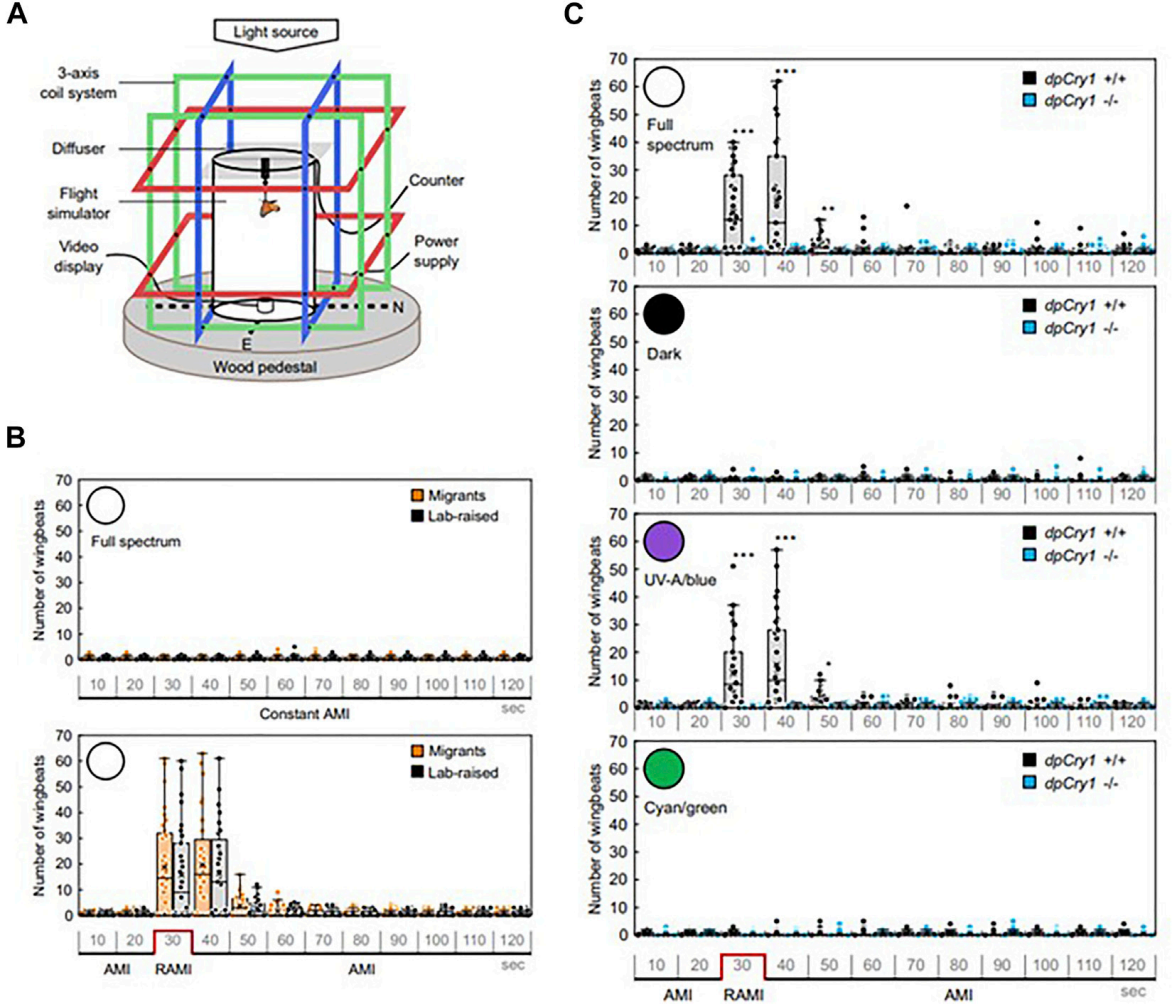
Magnetic field effects on Monarch butterfly behaviour. **(A)** Flight simulator with three different axes of Helmholz coils with a Monarch tethered in the middle of the arena. **(B)** Migrant and lab-reared Monarchs do not respond to a constant ambient MF (∼45 μT AMI, top panel) unless it is reversed (RAMI, bottom panel), when they immediately increase their wingbeats in response in full spectrum light. **(C)** The wingbeat response to RAMI is observed under full spectrum light but not in darkness in wild-type of *DpCry1* ^−/−^ knockout Monarchs. Neither does *DpCry1* ^*−/−*^ show the response in blue/UV light in contrast to the wild-type. Neither wild-type nor the mutant respond to RAMI under cyan/green light (480–580 nm) (Figure redrawn from Figures 1, 2 of Wan et al., 2021 under CC open access (http://creativecommons.org/publicdomain/zero/1.0/). See article for description of statistics).

While there are several examples of magnetic field effects on behaviour in insects only in a few have any kind of genetic manipulation been attempted. The Monarch butterfly *D. plexippus* provides a spectacular example of migration from the northern parts of North America to Mexico and back and is therefore the insect of choice for studying long-distance migration (Reppert, et al., 2010). Among various geophysical cues used for navigation, Monarchs also use the earth’s magnetic field as an inclination compass as demonstrated using flight simulators under UV and blue light (Guerra, et al., 2014). Using a flight simulator with three sets of Helmholtz coils so that inclination, declination and intensity of the MF could be manipulated (Figure 4A) it was also observed that reversing the natural magnetic inclination increased the butterfly’s wingbeat frequency under 380–430 nm but not 480–580 nm wavelengths (Wan et al., 2021) (Figure 4B). Gene editing of *DpCry1* but not *DpCry2* obliterated the magnetically-induced phenotype and covering the eyes or antennae with black paint impaired the wingbeat MF responses (Figure 4C). The fact that *Dp*CRY2 is dispensable for the MF phenotype is inconsistent with some of the reports in *Drosophila* in which *Dp*CRY2 shows MF effects. However, the manipulations employed here were not the typical GAL4-mediated overexpression studies used in *Drosophila*, but mutations generated by CRISPR-Cas9 editing. Also, the MF employed was similar to the earth’s, not several or many times more intense as usually (but not always) used in flies. These caveats should be taken into account when attempting to interpret the biological significance of MF effects in *Drosophila* behaviour, although they do not invalidate the fact that MF effects can be observed reliably in flies.

**FIGURE 5:**
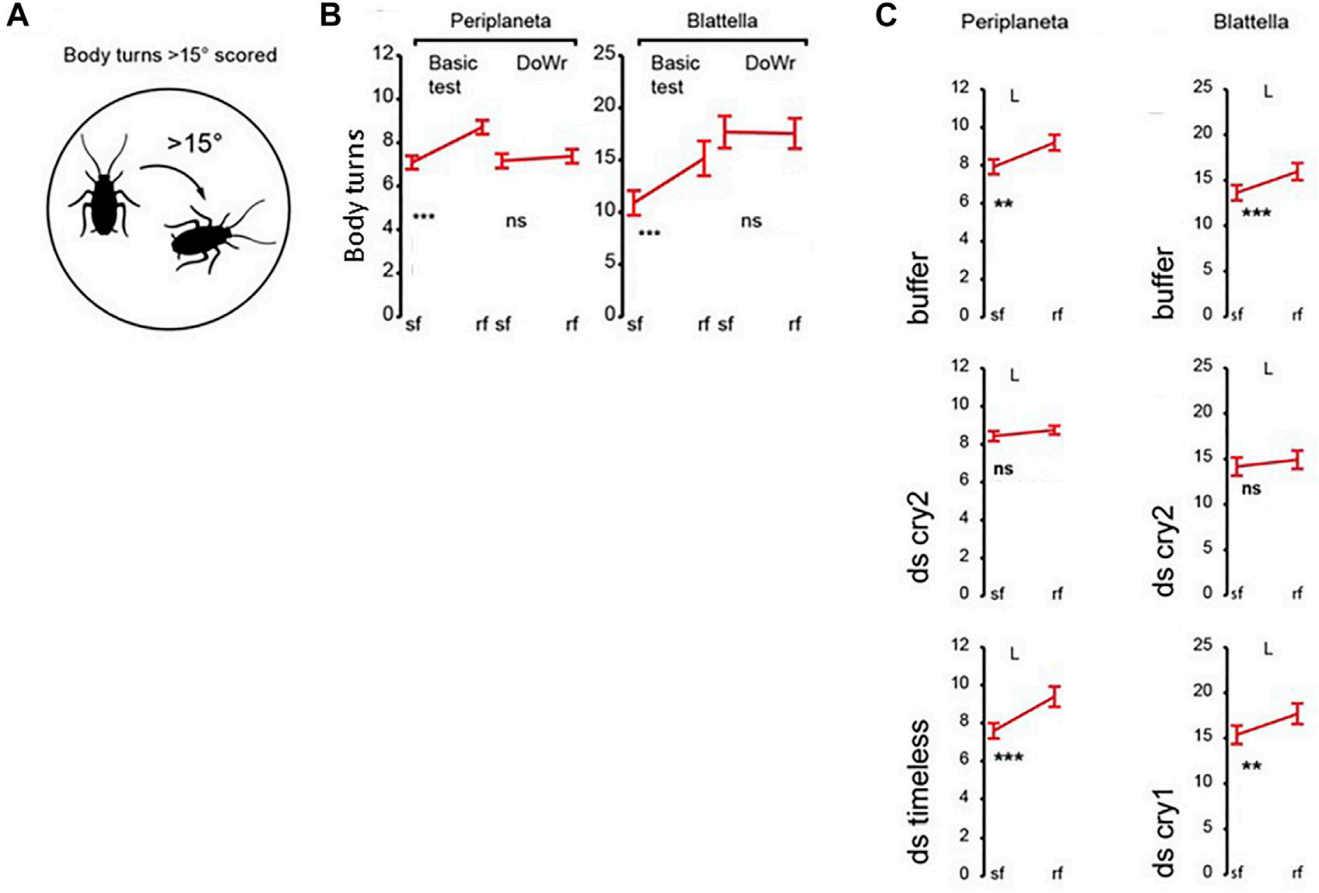
Genetic analysis of magnetosensitivity in cockroaches (Bazalova et al., 2016). **(A)** Diagrammatic representation of the body turn phenotype in response to a rotating MF. **(B)** Body turns in response to a MF (∼45μT, 18 μT horizontal component) for *Periplaneta americana* and *Blattella germanica*. Sf, steady field, rf rotating field. Basic test—MF exposure, DoWr-sham double wrapped coils. Under a MF (Basic) a significant increase in turning is observed in a rotating field (rf) for both species. Y-axis represents body turns >15^o.^ **(C)**. Transient double stranded (ds)RNAi knockdown of *Pacry2* and *BgCry2* but not *Bgcry1* nor *Patimeless (Patim)* eliminates the MF-mediated turning response. For **(B,C)** **p < 0.01, ***p < 0.001. Figure redrawn and simplified from Figures 1–3 from Bazalova et al (2016) with permission.

In the cockroach, *Periplaneta americana,* a rotation of the magnetic field generates body turns, which are eliminated in a dsRNAi knockdown of *Pacry2*, the only CRY encoded in the genome (Figures 5A,B). Disrupting circadian locomotor activity rhythms either with constant light or with *Patimeless* knockdown, failed to disturb the MF phenotype reflecting its independence from the circadian clock (Bazalova et al., 2016) (Figure 5B). In another cockroach species, *Blattella germanica,* which encodes both vertebrate-like CRY2 and *Drosophila*-like type 1 CRY, somewhat surprisingly, only knockdown of *Bg*CRY2 disrupted the MF-mediated turning response (Figure 5C). In the latter species, the MF phenotype was observed under wavelengths corresponding to UV through to blue-green but there was a precipitous drop off in effect from 505 to 528 nm, consistent with CRY spectral sensitivity (Bazalova et al., 2016). Covering the eyes with black but not transparent paint disrupted the MF effect in *P. americana* and immunocytochemistry revealed that *Pa*CRY2 was expressed beneath the retina, probably in glial cells sandwiched between two basement membranes. By being immobilised in this way, *Pa*CRY2 could potentially serve as a direction sensor. Clearly, the focus on the type 2 CRYs here is at odds with the Monarch study outlined above and again underscores once again that the ability of CRY2 molecules to bind FAD effectively may change according to species-specific sequences and cellular environments.

An aversion paradigm where an insect ‘freezes’ when stimulated with a burst of hot air, paired with exposure to a rotating MF at a 505 nm wavelength, was used in the firebug *Pyrrhocoris apterus*. Males but not females showed the conditioned response to a MF and this was disrupted by CRISPR-Cas9 editing of the insect’s *PyaCry2* gene. An unexpected result was that a modest MF response could still be generated even after the animals had been left for 24 h (but not 48 h) in complete darkness after the initial training (Netušil et al., 2021). This is particularly surprising, because the canonical RPM requires light to generate the FAD^o−^ Trp^o+^ radical pair. The implication here is that light activation of *Pya*CRY2 ignites a chain of events resulting in magnetosensitive RPs that are detectable for more than 20 h. It is highly unlikely that an intramolecular FAD^o−^ Trp^o+^ radical pair, may be stable for so long, suggesting that alternative magnetosensitive RPs can occur and operate both in parallel and sequentially within cells. There is some precedent for MF effects occurring after short periods of darkness in both birds (Wiltschko et al., 2016) and plants (Pooam et al., 2018; Hammad et al., 2020). Although such effects occur after tens of minutes of darkness at most, it is still difficult (if not impossible) to incorporate them into a canonical RPM. These ‘dark’ *P. apterus* results therefore generate a further conundrum for the classic RPM but the modest nature of the effects suggest that independent replication in this or other species would be helpful.

### Discussion, speculation and conclusions

The canonical RP hypothesis suggests that the effect of a MF on CRY involves stabilizing the light-induced intramolecular RP. This is because the MF ultimately would decrease the likelihood that the electron acquired by FAD^o−^ through photoactivation may migrate back to the terminal Trp^o+^ (Hore and Mouritsen, 2016) (Figure 1A). The electric charges of FAD^o−^ and Trp^o+^ cause strong intramolecular electrostatic interactions that are believed to modify light-activated CRY into a more ‘open’ conformation compared to its dark state, which is more amenable to protein-protein interactions for initiating a signalling cascade (Zoltowski et al., 2011; Czarna et al., 2013; Levy et al., 2013). Consequently, the MF would prolong the activation of a signalling pathway such that it would reach a threshold and transduce magnetoreception into complex behaviours like navigation and migration. Under a strict interpretation of such a scenario, natural selection would be expected to optimise the CRYs involved in navigation to become better sensors. This is the rationale of a recent and elegant *in vitro* spectroscopic study that compared the magnetic properties of non-migratory chicken and pigeon CRY4, to the migratory Robin (*Erithacus rubecula*) *Er*CRY4 (Xu et al., 2021). The results revealed that *Er*CRY4 is more magnetically sensitive than CRY4 in the other two species, and that the four Trps of the donor chain were important for magnetic properties as revealed by using Trp-to-Phe mutants. *Er*CRY4 and the FAD^o−^ Trp^o+^ radicals thus fulfilled the physico-chemical requirements of an optimised magnetosensor. However, a ‘fly in the ointment’ was that one Trp mutant generated a more sensitive magnetic field effect than wild-type *Er*CRY4, which is puzzling. Additionally, the study did not address a number of biological findings that are not easy to reconcile with the canonical FAD^o−^ Trp^o+^ RP mechanism proposed to underlie the magnetosensitivity of *Er*CRY4, for example, studies, in *Arabidopsis* and *E. rubecula* which suggest that reoxidation in darkness may provide the magnetically sensitive RPs (Müller and Ahmad, 2011; Wiltschko et al., 2016; Pooam et al, 2018; Hammad et al., 2020).

The studies performed on insects have been important for discovering the wide range of behaviours that, under appropriate conditions, are sensitive to MFs. These phenotypes rely on different neuronal circuits, so one conclusion is that magnetosensitivity may be a general cellular property, and not just of excitable cells, given the plant literature on MFs. Therefore, it is likely that navigation did not require the evolution of specialised *ad hoc* proteins and signalling pathways but the organisation of general properties into a sensory cascade. Additionally, we have learned that while magnetosensitive RPs are likely sensors of MFs in biological systems, there may be more than one type of magnetosensitive RP, not just the light-activated CRY FAD^o−^ Trp^o+^ (eg Müller and Ahmad, 2011). Radicals are very reactive chemical species and hence short-lived so light-generated RPs cannot persist for tens of minutes (as magnetosensitivity in plants and birds suggest) or many hours (as suggested in *P. apterus*) in darkness. The necessary conclusion from these experiments is that at least in some cells the activation of CRY by light ignites chain-reaction mechanisms that can persist for some time in darkness generating alternative, short-lived magnetosensitive RPs. One example is the mechanism of reoxidation of fully reduced FADH^−^ into oxidised FAD by O_2_ that operates both in light and in darkness (Ritz et al., 2009; Müller and Ahmad, 2011). In the same cell, different magnetosensitive RPs may be present in parallel (perhaps even having a synergistic effect) and sequentially, as some RPs do not require the immediate presence of light for their generation.

This brings us to consider the possible mechanisms by which magnetoreception is transduced into behaviour. The first candidate model is the prolonged signalling cascade described above that follows a change in CRY conformation that is central to the canonical RP hypothesis. However, an alternative (or additional) mechanism stems from the redox properties of FAD^o−^ (and potentially of Trp^o+^ see Hong et al., 2018). As mentioned earlier full-length *d*CRY has optogenetic properties. When *d*CRY was expressed in olfactory neurons that are not normally light-sensitive, a similarly enhanced neuronal firing phenotype was observed under blue light. Pharmacological blockade of K^+^ but not Na^+^ channels attenuated the response to blue light in clock neurons (Fogle et al., 2011). Furthermore, in *hyperkinetic* (*hk*) null mutants lacking the potassium Kvβ subunit this response was reduced. Structural and functional analyses suggest that Kvβ channels are redox responsive, as they harbour the redox sensitive cofactor NADPH/NADP^+^ that modulates their function. Indeed, altering the cellular redox environment changes the firing patterns of clock neurons in a HK-dependent manner. Additionally, in *hk* null mutants the blue light response of clock neurons can be rescued by wild-type but not by Kvβ variants in which the redox sensor has been impaired. Mutations in other K^+^ channel subunits such as EAG (Kvα) that normally co-assemble with HK also disrupted the *d*CRY neuronal response (Fogle et al., 2015).

Thus the potassium channel β subunit (Kvβ) HK (here we use the fly example for simplicity, but it is a general hypothesis) harbours NADPH and HK binds to the α subunits (Kvα) such as EAG, SHAKER (SK) and others regulating the opening of the composite channel according to the redox state of the NADPH/NADP^+^ cofactor. Consequently, the MF-dependent potentiation of the redox chemistry of FAD could generate a biological response (Arthaut et al., 2017) by requiring FAD^o−^ (hence CRY) to be localised close to NADPH (hence HK). A plasma membrane scaffold protein encoding several modular PDZ domains is Discs-large-1 (DLG1) and is located in the synaptic active zone. Several different classes of proteins including EAG, SH, and HK bind (directly or indirectly) to DLG1 facilitating signalling (Walch, 2013). Potentially, CRY through the PDZ binding motifs in its CTT could also interact with DLG1. Thus, CRY *via* its FAD and its CTT would act both as the sensor and the transducer, enhancing the formation of FAD^o−^ under MFs and then shepherding it to the membrane where it is converted into a change in ion flux (by affecting the opening of potassium channels). A major challenge here is that in several assays, the CRYCT (that contains the CTT) by itself can mediate magnetosensitivity even though it cannot bind FAD directly. Perhaps then, CRYCT is part of a ‘magnetoplex’ in which one or more components associate with FAD/flavoprotein while the CTT brings such a complex to the vicinity of the neuronal membrane? Under this scenario CRYCT is the transducer whereas the sensor is carried by FAD or another RP competent molecule. In this context, FAD has recently been shown to generate a magnetically sensitive intramolecular RP in HELA cells (Ikeya and Woodward, 2021) confirming *in vitro* studies that revealed magnetosensitivity in solution at physiological pH (Antill and Woodward, 2018). This may help explain why full length CRY encoding the Trp triad or tetrad is sufficient but not necessary to generate a magnetically sensitive RP because FAD may accomplish this alone under certain conditions (somehow with the help of the CRY-CT). Furthermore, in darkness CRY may also intervene as the transducer that could explain magnetosensitivity under non-photic environments.

By analogy, dCRY also plays a functional role in the visual signalling cascade, in addition to its cardinal roles in circadian light resetting and magnetosensitivity (Mazzotta et al., 2013; Mazzotta, et al., 2018; Schlichting et al., 2018). This is accomplished through the CaM binding region in the *d*CRYCT and the PDZ binding motifs in the CTT. *d*CRY associates with Inactivation No Afterpotential D (INAD), a scaffold protein encoding 5 PDZ domains that is located in the photoreceptors (Mazzotta et al., 2013). INAD is part of the ‘signalplex’, a complex of proteins that mediates phototransduction and several of its constituents, including INAD, have CaM binding domains. Thus, INAD has structural similarities to DLG1 and it is likely that *d*CRY, through PDZ binding and using CaM as a bridge, is recruited by INAD to contribute to the visual signalplex of the fly (Mazzotta et al., 2018). A magnetosensitive *d*CRY acting within this complex in the retina has been speculated to have the potential to allow flies to ‘see’ the MF (Foley et al., 2011) in the same way as it was been suggested that birds may also ‘see’ the MF lines of inclination (Ritz et al., 2000; Stoneham et al., 2012).

In summary, using different organisms including our favourite, *D. melanogaster*, we provide an alternative view that builds on the classic formulation of the RPM, but extends it so that it can incorporate some of the more conflicting results within the field. We also extend the RP scenario to the putative downstream effectors, such as DLG1 and HK, that can potentially alter behaviour under a MF. Undoubtedly, the genetic dissection of magnetic phenotypes *in vivo,* combined with physical-chemistry *in vitro,* will continue to stimulate (and puzzle) workers in this area, but ultimately, it will extend our understanding of this remarkable sensory modality.

## Data Availability

The original contributions presented in the study are included in the article/supplementary materials, further inquiries can be directed to the corresponding author.
